# US adult smokers’ perceived relative risk on ENDS and its effects on their transitions between cigarettes and ENDS

**DOI:** 10.1186/s12889-022-14168-8

**Published:** 2022-09-19

**Authors:** Sooyong Kim, Saul Shiffman, Mark A. Sembower

**Affiliations:** PinneyAssociates Inc, 201 N. Craig St, Ste 320, Pittsburgh, PA 15213 USA

**Keywords:** Electronic cigarette, Electronic nicotine delivery system, Tobacco, Smoking, Cigarette, Risk perception, Harm reduction

## Abstract

**Background:**

Perceived risk reduction motivates smokers to switch to electronic nicotine delivery systems (ENDS). This research examines US smokers’ relative risk perceptions and their prospective association with various behavioral stages of switching to ENDS.

**Methods:**

Data from the nationally representative, longitudinal Population Assessment of Tobacco and Health (PATH) Adult survey, Waves 1 (2014) through 5 (2019), were analyzed. We assessed the association between the perceived risk of ENDS relative to cigarettes (“less harmful” vs. “equally harmful” or “more harmful”) and 1) adoption of ENDS (among never-ENDS-using smokers), 2) complete switching to ENDS (i.e., stopping smoking, among ever-ENDS-using smokers), and 3) avoiding reversion to smoking (among smokers who had switched to ENDS), at the next wave.

**Results:**

The proportion of US smokers perceiving ENDS as less harmful than cigarettes continually decreased, reaching 17.4% in Wave 5 (2019). Current smokers with such belief were more likely to adopt ENDS (aOR 1.31; 95% CI 1.15–1.50) and switch completely to ENDS (aOR 2.24; 95% CI 1.89–2.65) in the subsequent wave. Among smokers who had switched within the past year, such beliefs predicted avoidance of resumption of smoking in the next wave (aOR 0.55; 95% CI 0.33–0.93).

**Conclusions:**

Smokers’ beliefs about the relative risk of ENDS compared to cigarettes had a strong and consistent association with transitions between smoking and ENDS use. Addressing the growing misperception about ENDS has the potential to contribute to public health by encouraging smokers’ switching to ENDS.

**Supplementary Information:**

The online version contains supplementary material available at 10.1186/s12889-022-14168-8.

## Background

Combustible cigarette smoking continues to be the leading cause of preventable morbidity and mortality in the US, [1] with 34.1 million US adults smoking in 2019 [2]. There is a scientific consensus that the toxicity of cigarettes largely comes from the products of combustion [3]. Accordingly, electronic nicotine delivery systems (ENDS), which deliver nicotine without combustion, have been estimated to have a considerably lower risk profile compared to cigarettes [1, 4–6]. This concept of the continuum of risk (that some nicotine-delivering products are less harmful than the others), while broadly acknowledged by the literature, regulatory agencies, and health bodies around the world, [4, 5, 7–12] has not been well-communicated to smokers or the general population. In fact, several studies suggest that the proportion of US adults who perceive ENDS as at least as harmful as cigarettes continues to increase, [13, 14] reflecting the exacerbation of this misunderstanding among the general public. We refer to such beliefs as misperceptions, consistent with the literature on the topic [4, 14, 15].

As suggested by the health belief model [16] and the theory of planned behavior, [17] perceived risks and benefits are major drivers of health behaviors. Current and former smokers who have used ENDS often indicate that the one of their most important reasons for using ENDS is because they perceive ENDS as posing lower health risks compared to cigarettes [18]. Conversely, misperceiving the relative risk of ENDS was one of the reasons given for not being willing to try ENDS and for choosing not to switch completely to ENDS [19]. Collectively, these results suggest that the misperception of ENDS may deter smokers from harm reduction behaviors such as adopting and switching to ENDS, [20, 21] and may lead to increased harm by driving exclusive ENDS users back to cigarette smoking [21].

For ENDS use to have its intended harm reduction benefit on smokers who would not otherwise quit, they must start using ENDS (which we refer to as “adoption”), stop smoking and switch instead to ENDS use (“switching”), and maintain the switched status and avoid reverting to smoking (“reversion”). Although dual use may still be beneficial if accompanied by a substantial reduction in cigarette consumption, [22] switching completely has the biggest potential benefit. The first year after switching was considered as a particularly important period of risk for reversion, as this is the highest risk period for relapse in smoking cessation [23, 24] and ENDS use [25, 26]. Using the Population Assessment of Tobacco and Health (PATH) data, the current research aims to examine the association of adult smokers’ relative risk perception of ENDS on future smoking/vaping behaviors. The analyses examine the prospective relationship between risk perceptions and three aspects or stages of switching to ENDS: (1) initial adoption of ENDS; (2) switching away from smoking to ENDS; and (3) reverting to smoking (vs. maintaining switching).

## Methods

### Sample

Data were from the PATH Adult study, an ongoing prospective, nationally representative cohort study of US adults’ tobacco use. The study recruitment was based on a multi-level probability sampling design with oversampling of several underrepresented subgroups. The complex survey design with population and replicate weights adjusts for the study design as well as survey non-response, which allows for generalizable estimation of tobacco use behaviors of US civilian, non-institutionalized adults. Details of the PATH study methodology have been published elsewhere [27]. The analysis combined PATH data from Wave 1 (collected between Sep 2013 – Dec 2014), Wave 2 (Oct 2014 – Oct 2015), Wave 3 (Oct 2015 – Oct 2016), Wave 4 (Dec 2016 – Jan 2018), and Wave 5 (Dec 2018 – Nov 2019) of PATH. The longitudinal data allow assessment of the temporal relationship; i.e., how risk perception at one wave (referred to as Wave t) is associated with the behavior in the next wave (Wave t + 1).

Participants were classified based on their smoking and vaping status at each wave. Based on the well-established definition used by the PATH study as well as other national surveys, [27–29] participants were defined as current established smokers if they had smoked 100 or more cigarettes in their lifetime and stated that they currently smoked “some days” or “every day.” Current established smokers were further stratified by lifetime history of ENDS use. Never-users of ENDS were the sample for analyses of ENDS adoption. For analyses of switching, the denominator was smokers who had used ENDS, whether they were currently using them or not. This encompasses all smokers who have completed the initial step towards switching and may achieve complete switching in the following wave, but does not specifically limit to those who were dual users at the time of the survey, as this would oversample persistent dual users and would fail to capture those who adopted ENDS and switched between survey waves. For analyses of reversion to smoking, the sample included former smokers who reported switching to exclusive ENDS use (i.e., using ENDS and no longer smoking).

Participants could have been included in the analysis more than once as long as they met the eligibility criteria for each particular analysis at the relevant wave. The generalized estimating equation (GEE) analysis took account of multiple observations per person, as well as accounting for missing observations at particular waves.

Initial univariate analyses used 9,321 observations from 4,842 never-ENDS-using smokers for the analysis of ENDS adoption; 22,920 observations from 9,438 ever-ENDS-using smokers for the analysis of switching to ENDS; 1,848 observations from 1,151 switchers for the analysis of reversion back to cigarettes. The number of observations used for multivariable modeling may differ due to occasional missing covariates and is shown in footnotes to the corresponding tables.

### Measures

#### Predictor: relative risk perception

Relative risk perception was assessed at each wave with a single question. Participants were asked, “Is using e-cigarettes less harmful, about the same, or more harmful than smoking cigarettes?” with participants choosing one of three options, “less harmful,” “about the same,” or “more harmful.” Consistent with previous analyses of risk perception in the PATH data, [14, 30, 31] responses were dichotomized into “less harmful than cigarettes (*correct perception*)” vs. “equally or more harmful than cigarettes (*misperception*).” Sensitivity analyses were conducted using all three levels of relative risk perception (Supplementary Table 1). No significant differences between perceiving ENDS to be “equally harmful” and “more harmful” than cigarettes were found, though the effects reported below were seen particularly for the contrast between “less harmful” and “more harmful.” At Wave 1, the risk perception item was asked only of those who had seen or heard of ENDS.

#### Outcomes: adoption, switching, and reversion

Consistent with the definition of current cigarette smokers/ENDS users described above, study outcomes were also based on using cigarettes or ENDS “every day” or “some day,” compared to using “not at all.” Three outcomes were examined: adoption of ENDS (using ENDS, among previous-wave smokers who have never used ENDS), switching away from smoking with ENDS (no longer smoking but using ENDS, among previous-wave smokers who had used ENDS), and reverting to smoking (return to smoking, whether along with ENDS or not, among “switchers”; i.e., previous-wave former smokers who were currently using ENDS. In each case, the outcome at Wave t + 1 was modeled prospectively as a function of risk perception at wave t. For example, among smokers who at Wave t had never used ENDS, adoption of ENDS at Wave t + 1 was modeled with Wave t risk perceptions.

#### Covariates

Six covariates were included to account for sociodemographic characteristics: race/ethnicity, age, sex, educational attainment, marital status, and household income (see Tables 1, 2, and 3). Covariates were time-varying, changing to reflect participants’ wave-to-wave changes in sociodemographic factors such as age and income level. Marital status was not asked in the Wave 1 survey; therefore, marital status at Wave 1 was extrapolated from Wave 2.

#### Sample descriptors

To characterize the sample, smoking status (daily/nondaily), average cigarettes per day (CPD), years of regular smoking, and time-to-first-cigarette (a measure of cigarette dependence [32]) were described for current smokers. For switchers, average CPD when smoking, years of regular smoking before quitting, and the duration of quitting were described (Supplemental Table 2). We did not include these as covariates in the model, as they could be affected by risk perceptions, leading to overcontrol in the models.

### Analyses

The relationship between the risk perception (at Wave t) and the outcome (at Wave t + 1) was assessed using weighted GEE models [33]. Each observation was weighted using survey weights at Wave t + 1. Weights could vary over waves, and took account of non-response at each wave, as well as entry of new participants into the PATH adult cohort. GEE models estimated the population-averaged effects of risk perception while accounting for the repeated-measure design and resultant within-participant interdependence in multi-wave data from the PATH study, using the SAS Macro developed by the PATH study team (see Kasza et al. [34]). Variances were estimated using Fay’s balanced repeated replication method with an adjustment factor of 0.3 [33]. An exchangeable correlation structure was used, based on the quasi-likelihood information criterion (QIC) statistics [35]. Multivariable models were adjusted for time (using the wave number), and the covariates described above. Based on the temporal patterns observed in Figs. 2, 3 and 4 and improvement in model fit, quadratic terms were included in the adoption and switching analyses; the fit indices for the reversion models did not indicate a quadratic term was needed in those models. Inclusion of the quadratic term did not change the association between risk perceptions and subsequent outcomes (inclusion of the quadratic term reduced the aOR for risk perceptions by no more than 0.05). Follow-up subgroup analyses were conducted for the outcome of reversion among switchers, to examine reversion in the first year after switching.

## Results

**Fig. 1:**
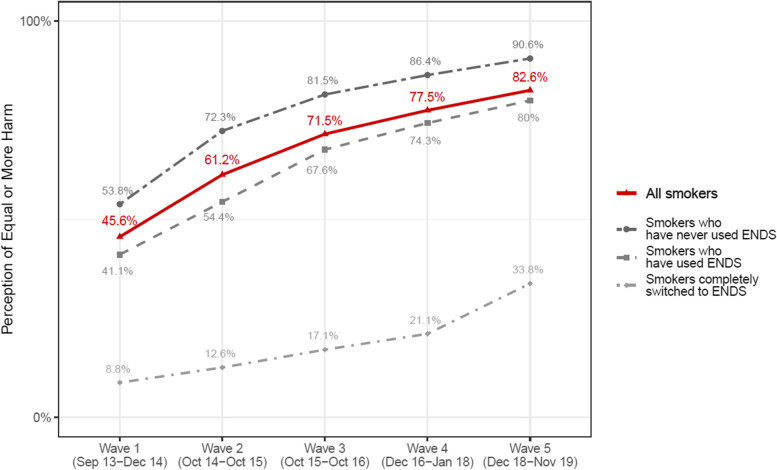
Percent of smokers perceiving ENDS to be at least as harmful as cigarettes. ENDS: electronic nicotine delivery system. Note: Statistics indicate weighted percentages, calculated cross-sectionally at each wave

**Fig. 2:**
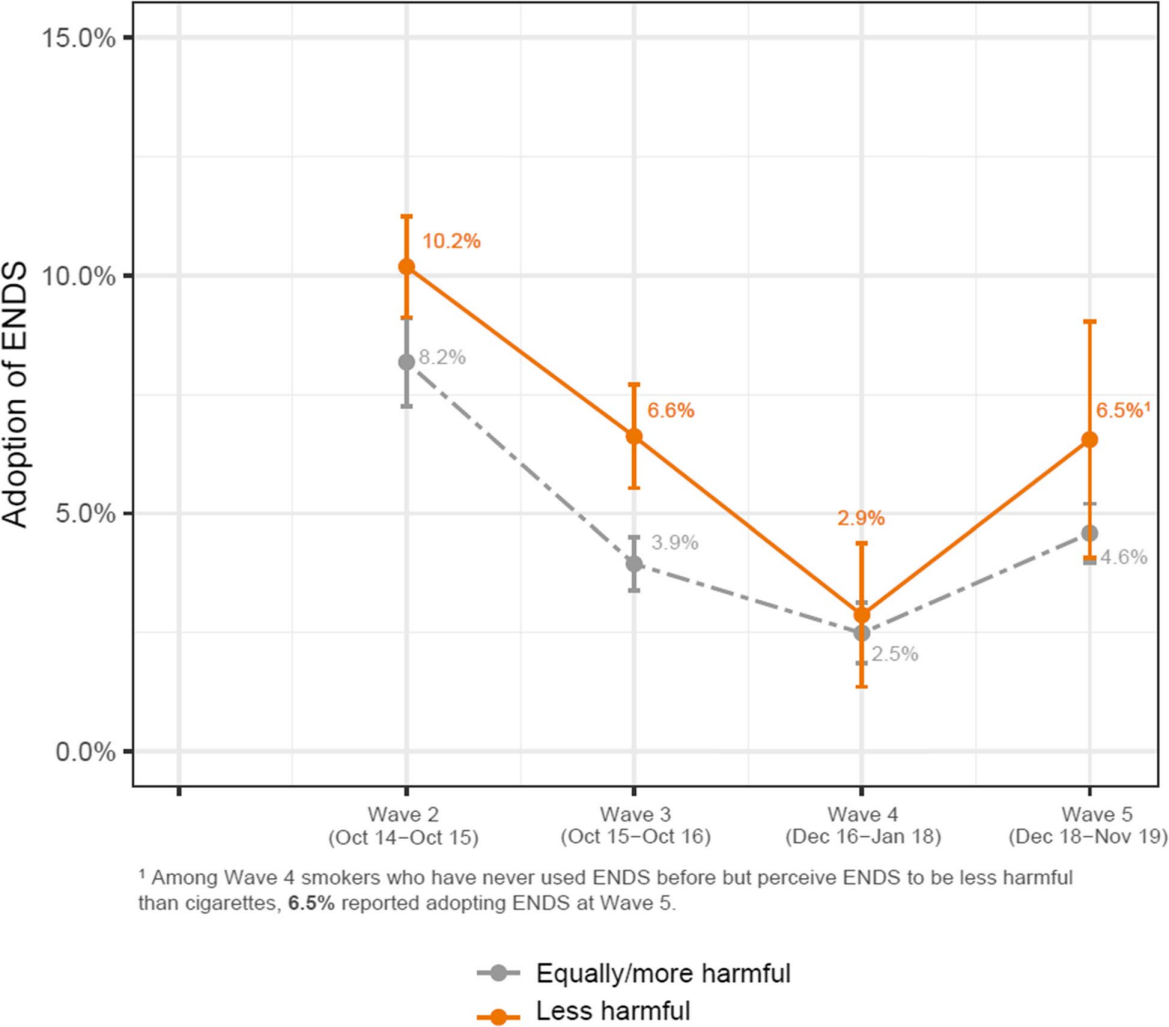
Percent of never-ENDS-using smokers adopting ENDS, by previous-wave relative risk perceptions. ENDS: electronic nicotine delivery system. Note: Weighted percentages and standard errors using ENDS at Wave t + 1, stratified by the relative risk perception on ENDS at Wave t, calculated cross-sectionally at each wave. Individual PATH participants could contribute to more than one time-point

**Fig. 3:**
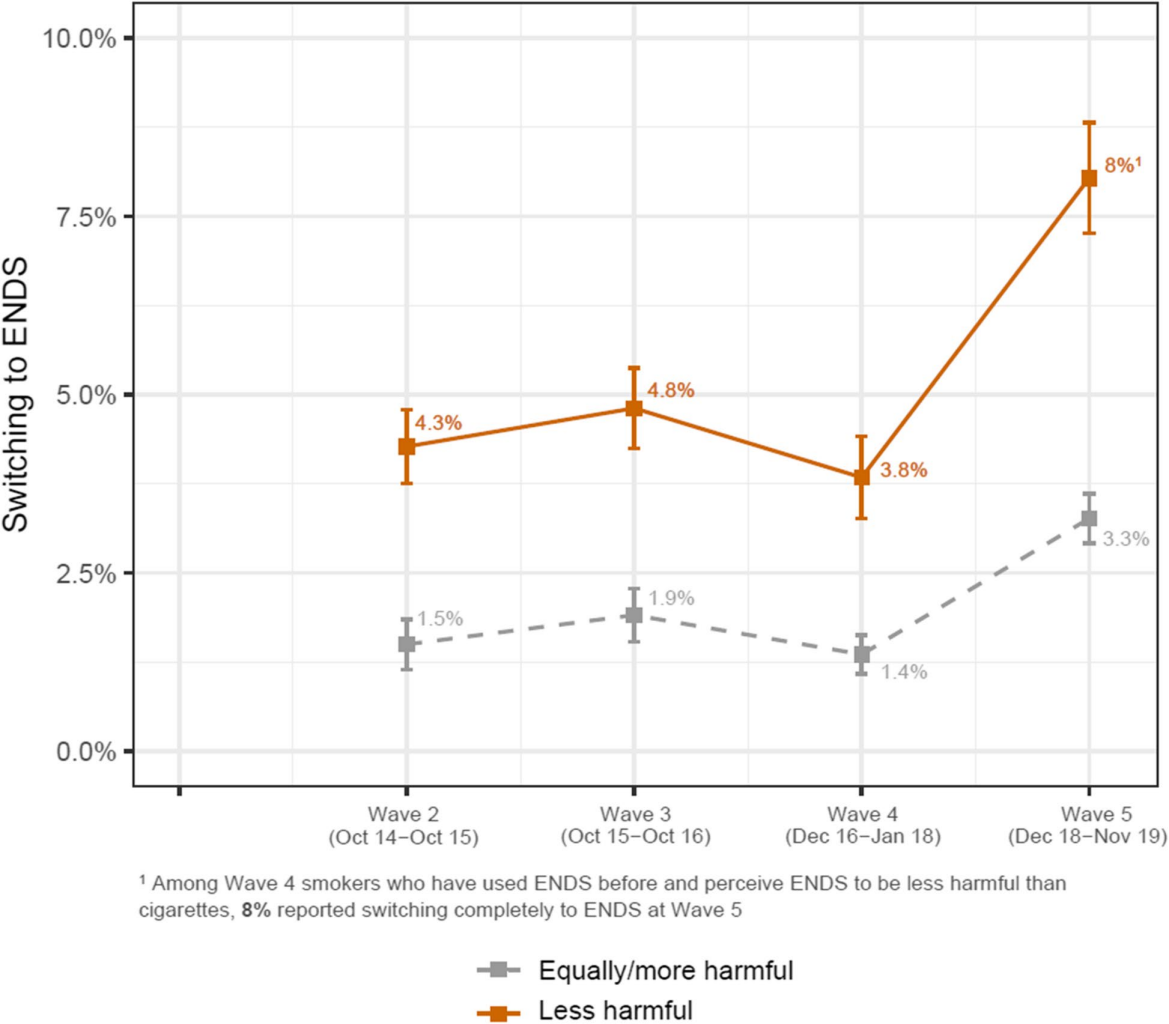
Percent of ever-ENDS-using smokers who switched from smoking to ENDS, by previous-wave relative risk perceptions. ENDS: electronic nicotine delivery system. Note: Weighted percentages and standard errors who switched away from smoking at Wave t + 1, stratified by the relative risk perception on ENDS at Wave t, calculated cross-sectionally at each wave. Individual PATH participants could contribute to more than one time-point

**Fig. 4:**
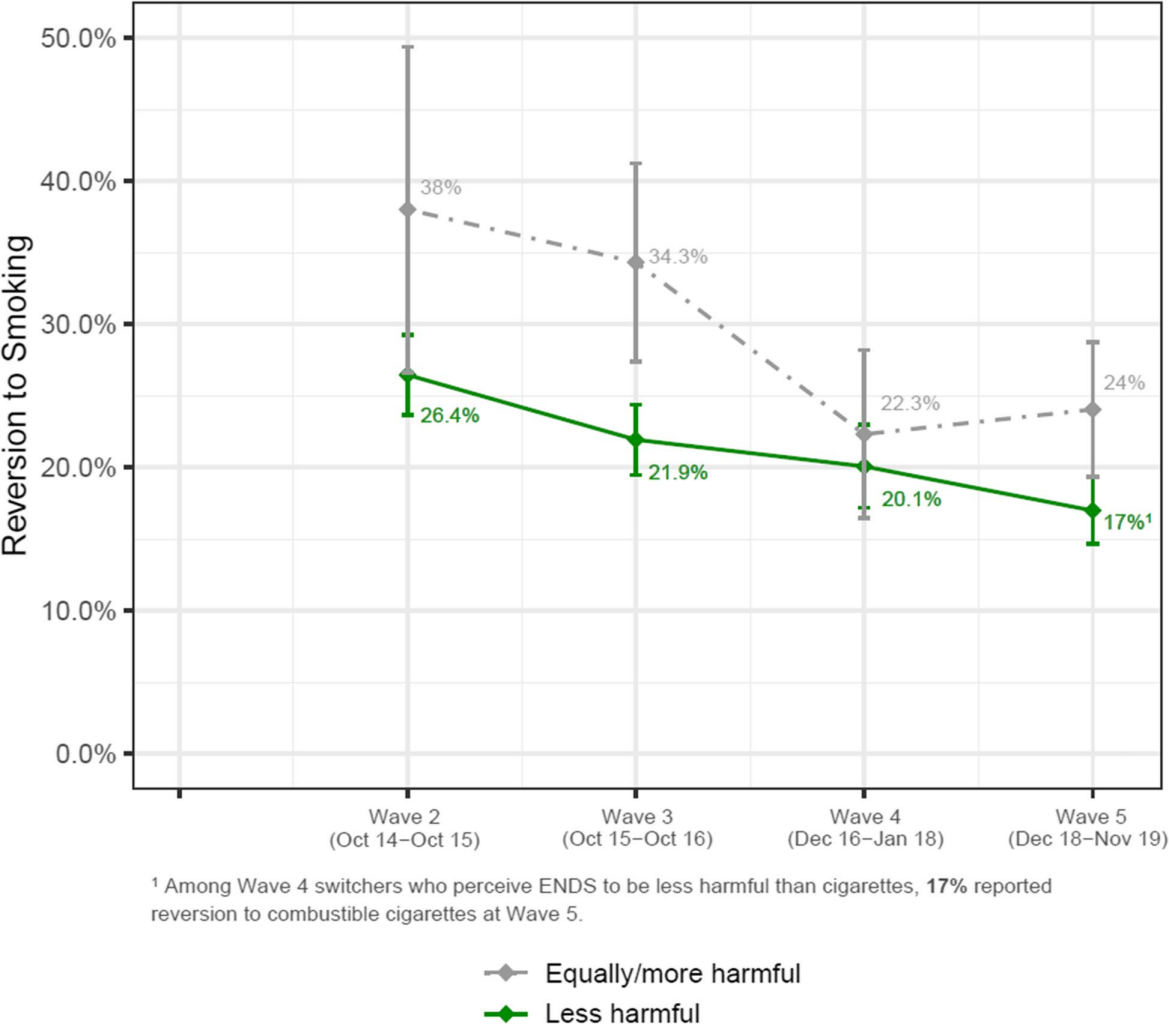
Percent of smokers who switched that reverted back to smoking, by previous-wave relative risk perceptions. ENDS: electronic nicotine delivery system; Switcher: former established smokers who are currently using ENDS. Note: Weighted percentages and standard errors of participants who reverted to smoking at Wave t + 1, stratified by the relative risk perception on ENDS at Wave t, calculated cross-sectionally at each wave. Individual PATH participants could contribute to more than one time-point

Figure 1 shows the trend of participants’ relative risk perception over Waves. The perceived risk of ENDS compared to smoking increased over time among current established smokers, with increasing proportions of smokers perceiving ENDS to be equally or more harmful than cigarettes (red line). In the subsamples of this study (greyscale lines), across Waves, Switchers were least likely to report perceiving ENDS as harmful as smoking, while smokers who had never used ENDS were most likely to report such perceptions. However, the proportion perceiving ENDS as at least as harmful as smoking increased over time in all groups, even among Switchers. Figures 2, 3, and 4 depict the proportion of adoption, switching, and reversion of the participants at the following wave by the level of risk perception, respectively, and the standard errors of the proportions.

For each of the following analyses Supplemental Table 3 summarizes the sociodemographic characteristics of the sample and their outcomes in the following wave.

### Smokers’ adoption of ENDS

As seen in Fig. 2, the likelihood of ENDS adoption varied significantly as a quadratic function of time: the odds of adoption decreased roughly until Wave 4, then increased between Waves 4 and 5. In univariate analysis, smokers who perceived ENDS as less harmful than cigarettes had 68% greater odds (OR = 1.68, 95% CI: 1.35–2.09) of reporting later adoption of ENDS (Table 1). After adjusting for these sociodemographic factors in Multivariable analyses, perceiving ENDS as less harmful than cigarettes was associated with 35% (aOR = 1.35, 95% CI: 1.14–1.50) increased odds of later adoption of ENDS.

**Table 1:** Adoption of ENDS among never-ENDS-using smokers: risk perceptions and sociodemographic characteristics

		**Smokers who have never used ENDS – Adoption**	
		**Univariate results**	**Multivariable results**^b^
		OR (95% CI)	aOR (95% CI)
*Time*	Linear	**0.19 (0.10 – 0.35)**	**0.22 (0.12 – 0.43)**
	Quadratic	**1.32 (1.16 – 1.51)**	**1.31 (1.14 – 1.50)**
*Risk perception*	Equally/more harmful	Reference	
	Less harmful	**1.68 (1.35 – 2.09)**	**1.35 (1.04 – 1.73)**
*Race/ethnicity*	NH White	Reference	
	NH Black	**0.60 (0.45 – 0.80)**	**0.58 (0.42 – 0.80)**
	Hispanic	**0.57 (0.40 – 0.82)**	**0.55 (0.37 – 0.81)**
	NH others	1.20 (0.72 – 2.02)	1.06 (0.64 – 1.78)
*Sex*	Male	Reference	
	Female	1.03 (0.80 – 1.34)	0.99 (0.76 – 1.28)
*Age*	18 – 24	Reference	
	25 – 44	**0.49 (0.35 – 0.68)**	**0.54 (0.38 – 0.77)**
	45 – 54	**0.33 (0.24 – 0.46)**	**0.36 (0.24 – 0.53)**
	55 or older	**0.18 (0.13 – 0.25)**	**0.19 (0.13 – 0.28)**
*Marital status*^a^	Married	Reference	
	D/S/W	1.07 (0.81 – 1.41)	1.27 (0.96 – 1.69)
	Never married	**1.39 (1.10 – 1.76)**	1.12 (0.85 – 1.46)
*Household income*	< $25 k	Reference	
	$25 k – $50 k	1.07 (0.84 – 1.38)	0.99 (0.76 – 1.29)
	> $50 k	1.05 (0.83 – 1.33)	0.95 (0.71 – 1.28)
*Educational attainment*	HS/GED or less	Reference	
	Some college	1.23 (0.97 – 1.56)	1.08 (0.85 – 1.38)
	Bachelor or higher	1.05 (0.76 – 1.44)	0.92 (0.63 – 1.33)

Boldface represents statistically significant results

*NH* Non-Hispanic, *HS* High school, *D/S/W* Divorced/Separated/Widowed

^a^Marital status of Wave 1 has been extrapolated from the marital status of Wave 2 due to unavailability in the survey

^b^Multivariable results are based on 8480 observations from 4521 unique participants, and represent an analysis with all listed variables simultaneously in the model

### Smokers’ switching to ENDS

As with the adoption of ENDS, the data showed a sharp rise in rates of switching between Waves 4 and 5 (Fig. 3). Univariate analyses showed that smokers who perceived ENDS as less harmful than cigarettes were more likely to subsequently switch to ENDS; their odds of switching in the following year were 134% higher (OR = 2.34, 95% CI: 2.00–2.76) than those of participants who thought ENDS were at least as harmful as cigarettes (Table 2). The association of switching with risk perceptions remained strong (a 127% increase in odds of switching, aOR = 2.27, 95% CI: 1.92–2.68) even after adjusting for demographic factors and the PATH wave.

**Table 2:** Switching among ever-ENDS-using smokers: risk perceptions and sociodemographic characteristics

		**Smokers who have used ENDS – Switching**	
		**Univariate results**	**Multivariable results**^b^
		OR (95% CI)	aOR (95% CI)
*Time*	Linear	**0.43 (0.25 – 0.73)**	**0.49 (0.29 – 0.83)**
	Quadratic	**1.20 (1.08 – 1.34)**	**1.20 (1.08 – 1.34)**
*Risk perception*	Equally/more harmful	Reference	
	Less harmful	**2.34 (2.00 – 2.76)**	**2.27 (1.92 – 2.68)**
*Race/ethnicity*	NH White	Reference	
	NH Black	**0.52 (0.37 – 0.75)**	**0.63 (0.46 – 0.87)**
	Hispanic	**0.69 (0.51 – 0.93)**	**0.72 (0.53 – 0.97)**
	NH others	0.89 (0.63 – 1.26)	0.72 (0.51 – 1.02)
*Sex*	Male	Reference	
	Female	**0.73 (0.62 – 0.86)**	**0.85 (0.72 – 1.00)**
*Age*	18 – 24	Reference	
	25 – 44	**0.50 (0.41 – 0.61)**	**0.47 (0.37 – 0.60)**
	45 – 54	**0.28 (0.20 – 0.39)**	**0.29 (0.20 – 0.42)**
	55 or older	**0.40 (0.30 – 0.53)**	**0.41 (0.29 – 0.59)**
*Marital status*^a^	Married	Reference	
	D/S/W	**0.66 (0.51 – 0.85)**	0.84 (0.65 – 1.09)
	Never married	**1.35 (1.10 – 1.67)**	1.10 (0.87 – 1.39)
*Household income*	< $25 k	Reference	
	$25 k – $50 k	**1.49 (1.17 – 1.91)**	**1.36 (1.06 – 1.74)**
	> $50 k	**2.03 (1.64 – 2.51)**	**1.62 (1.30 – 2.03)**
*Educational attainment*	HS/GED or less	Reference	
	Some college	**1.76 (1.45 – 2.14)**	**1.54 (1.26 – 1.88)**
	Bachelor or higher	**2.28 (1.70 – 3.04)**	**1.87 (1.36 – 2.58)**

Boldface represents statistically significant results

*NH* Non-Hispanic, *HS*, High school, *D/S/W* Divorced/Separated/Widowed

^a^Marital status of Wave 1 has been extrapolated from the marital status of Wave 2 due to unavailability in the survey

^b^Multivariable results are based on 20,981 observations from 8941 unique participants, and represent an analysis with all listed variables simultaneously in the model

### Switchers’ reversion to smoking

In univariate analyses, participants who believed that ENDS were equally or more harmful than smoking had 37% higher odds (OR = 1.37, 95% CI: 0.98–1.93) of subsequently resuming cigarette smoking, though the association was not statistically significant (*P* = 0.0672). Controlling for these demographic factors did not materially change the findings (Table 3).

**Table 3:** Reversion to smoking among switchers: risk perceptions and sociodemographic characteristics

		**Switchers – Reversion**	
		**Univariate results**	**Multivariable results**^d^
		OR (95% CI)	aOR (95% CI)
*Time*	Linear^a^	**0.88 (0.79 – 0.98)**	**0.87 (0.78 – 0.98)**
*Risk perception*	Less harmful^b^	Reference	
	Equally/more harmful	1.37 (0.98 – 1.93)^†^	1.34 (0.93 – 1.93)
*Race/ethnicity*	NH White	Reference	
	NH Black	0.87 (0.43 – 1.77)	0.79 (0.39 – 1.61)
	Hispanic	**1.61 (1.07 – 2.40)**	1.44 (0.96 – 2.16)
	NH others	1.16 (0.67 – 2.00)	0.98 (0.56 – 1.72)
*Sex*	Male	Reference	
	Female	0.96 (0.73 – 1.26)	0.96 (0.73 – 1.27)
*Age*	18 – 24	Reference	
	25 – 44	**0.57 (0.40 – 0.79)**	**0.63 (0.41 – 0.96)**
	45 – 54	**0.46 (0.29 – 0.74)**	**0.49 (0.29 – 0.80)**
	55 or older	**0.36 (0.23 – 0.56)**	**0.40 (0.23 – 0.68)**
*Marital status*^c^	Married	Reference	
	D/S/W	1.29 (0.86 – 1.93)	1.32 (0.86 – 2.04)
	Never married	**1.57 (1.19 – 2.07)**	1.10 (0.78 – 1.53)
*Household income*	< $25 k	Reference	
	$25 k – $50 k	**0.59 (0.40 – 0.88)**	**0.64 (0.42 – 0.96)**
	> $50 k	**0.62 (0.45 – 0.87)**	0.73 (0.51 – 1.06)
*Educational attainment*	HS/GED or less	Reference	
	Some college	1.07 (0.81 – 1.41)	1.07 (0.80 – 1.44)
	Bachelor or higher	0.69 (0.46 – 1.03)	0.80 (0.50 – 1.26)

Boldface represents statistically significant results

*NH* Non-Hispanic, *HS* High school, *D/S/W* Divorced/Separated/Widowed

^a^No quadratic effects of time were found at both univariate (OR 1.00; CI 0.88 – 1.13) and multivariate (OR 1.00; CI 0.87 – 1.14) level models

^b^For reversion analysis, the “less harmful” level of risk perception was used as the referent category

^c^Marital status of Wave 1 has been extrapolated from the marital status of Wave 2 due to unavailability in the survey

^d^Multivariable results are based on 1693 observations from 1063 unique participants, and represent an analysis with all listed variables simultaneously in the model

^†^Marginally significant at *P* = 0.0672

To further explore the relationship between risk perceptions and reversion, a further analysis stratified switchers according to the length of smoking abstinence (switching), as those with shorter switching history were expected to be more susceptible to reversion. Although the interaction between switch duration (less than a year vs. a year or more) and risk perceptions was not statistically significant (*P* = 0.5229), the literature on smoking cessation strongly suggests that the first year of switching would be a critical period in reversion to smoking, [23–26] suggesting the utility of examining the effect among those with shorter switch periods. Those who had switched for less than a year had significantly increased odds of reversion (by 78%, aOR = 1.78, 95% CI: 1.04–3.06) if they believed ENDS use was at least as harmful as smoking (Table 4). Though the effect was in the same direction for the participants who had switched for a year or more, it was smaller and not statistically significant.

**Table 4:** Reversion to smoking among switchers, stratified by duration of switching: risk perceptions and sociodemographic characteristics

**By Switch duration**		**Less than a year**	**A year or more**^d^
		*Multivariable results*^e^ *aOR (95% CI)*	*Multivariable results*^e^ *aOR (95% CI)*
*Time*	*Linear*^a^	0.89 (0.77 – 1.03)	0.94 (0.70 – 1.27)
*Risk perception*	Less harmful^b^	Reference	Reference
	Equally/more harmful	**1.78 (1.04 – 3.06)**	1.30 (0.70 – 2.43)
*Race/ethnicity*	NH White	Reference	Reference
	NH Black	0.59 (0.22 – 1.59)	0.63 (0.19 – 2.09)
	Hispanic	1.24 (0.75 – 2.04)	2.14 (0.79 – 5.83)
	NH others	1.31 (0.73 – 2.35)	0.27 (0.05 – 1.62)
*Sex*	Male	Reference	Reference
	Female	0.91 (0.65 – 1.27)	0.87 (0.48 – 1.59)
*Age*	18 – 24	Reference	Reference
	25 – 44	0.93 (0.54 – 1.58)	**0.25 (0.11 – 0.57)**
	45 – 54	0.80 (0.44 – 1.44)	0.34 (0.11 – 1.03)
	55 or older	0.81 (0.41 – 1.59)	**0.23 (0.07 – 0.71)**
*Marital status*^c^	Married	Reference	Reference
	D/S/W	0.80 (0.47 – 1.38)	**2.62 (1.08 – 6.38)**
	Never married	0.86 (0.57 – 1.31)	1.48 (0.79 – 2.76)
*Household income*	< $25 k	Reference	Reference
	$25 k – $50 k	0.86 (0.54 – 1.39)	0.47 (0.19 – 1.18)
	> $50 k	0.69 (0.45 – 1.04)	0.80 (0.39 – 1.62)
*Educational attainment*	HS/GED or less	Reference	Reference
	Some college	0.87 (0.57 – 1.34)	**2.12 (1.10 – 4.10)**
	Bachelor or higher	0.68 (0.37 – 1.27)	1.02 (0.44 – 2.37)

Boldface represents statistically significant results

*NH* Non-Hispanic, *HS* High school, *D/S/W* Divorced/Separated/Widowed

^a^No quadratic effects of time were found at either subgroup (OR 0.99, CI 0.84 – 1.16 among switchers with quit duration of less than a year; OR 1.09, CI 0.80 – 1.50 among switchers with quit duration of a year or more)

^b^For reversion analysis, the “less harmful” level of risk perception was used as the referent category

^c^Marital status of Wave 1 has been extrapolated from the marital status of Wave 2 due to unavailability in the survey

^d^Switching history of this subset is as follows (in years): Mean = 4.4; Median = 2.5; Interquartile range = 1.6 – 4.0

^e^The subset with less than a year duration of switching consists of 982 observations from 771 unique participants. The subset of participants who have switched a year or more are based on 959 observations from 519 unique participants. Some participants are included in both subsets as their switching history increases over time. Multivariable results represent an analysis with all listed variables simultaneously in the model

The interaction term (risk perception*switch duration) was not statistically significant (*P* = .5229)

## Discussion

Switching completely to ENDS has been suggested as a potentially effective harm reduction strategy for smokers who would not otherwise quit smoking cigarettes in the near term [6, 7, 10, 36]. Multiple behavioral theories predict that switching from smoking to ENDS would be promoted when smokers believe that ENDS use is less harmful than smoking, and would be suppressed if smokers believe otherwise [16, 17]. Our analyses confirm this association over multiple waves of the PATH survey. Smokers who believed ENDS were less harmful than smoking were significantly more likely to start using ENDS a year later, and also more likely to stop smoking and switch completely to ENDS. Additionally, the belief that ENDS are less harmful than smoking was associated with maintaining switching, that is, avoiding reversion to smoking among switchers who had been switched for less than a year. Taken together, the findings show that beliefs about the relative harms of ENDS compared to smoking appear to be strongly associated with harm-reduction behaviors, from adoption of ENDS, to switching to ENDS, to maintaining switched status over the first year of switching. While the correlational nature of the data precludes strong causal inferences, the data suggest that risk perceptions may influence harm reduction behaviors.

The results of this analysis are consistent with previous studies of smokers showing how risk perceptions affect smoking and ENDS use. As most smokers acknowledge the risk of cigarette smoking, [37] perceived health benefits are often one of the most important motivations for smokers to use ENDS [18, 38]. Indeed, those who perceive ENDS to be less harmful than cigarettes are more likely to seek information on ENDS, [39] adopt ENDS, [40–42] and ultimately switch completely from cigarettes to ENDS [21, 30, 43]. Conversely, misperception that ENDS are at least as harmful as smoking often emerges as an important reason for not trying ENDS, [19] stopping ENDS use even if they have used ENDS, [38] and maintaining dual-use rather than switching to exclusive ENDS use [19].

The present analysis supports these previous findings in a nationally-representative prospective sample, and demonstrates consistent relationships between risk perceptions and multiple stages of behavior change among smokers: in the adoption of ENDS, switching away from smoking with ENDS, and maintaining switched status (at least among recent switchers). Even after completing the full transition from smoking to exclusive ENDS use – which itself was associated with a favorable view of the relative risks of ENDS – believing that ENDS are at least as harmful as cigarettes was associated with switchers reverting back to smoking. That the significant association of risk perceptions with reversion to smoking was seen primarily among recent switchers should not be surprising. Although data on reversion to smoking over time among smokers who switched to ENDS is limited, reversion to smoking (i.e., relapse) after smoking cessation is much more likely in the first year after quitting [23, 24]. Similarly, the pattern of switching may be sufficiently well-established after a year to not be as influenced by risk perceptions.

The misperception that ENDS are at least as harmful as cigarettes has been growing over time [13, 14]. Factors such as misperception of the risks of nicotine, uncertainties about the long-term health effects of ENDS, a confounding of absolute vs. relative risk in scientific publications, media and public perceptions, media coverage, and specific campaigns emphasizing the potential risks of ENDS, often without contextual relative-risk information, have likely contributed to this trend [13, 14, 44]. The shock of EVALI and its initial misattribution to nicotine-containing e-cigarettes in mid-2019, was especially detrimental for the public’s harm perception [45, 46]. Given the relationship between favorable relative-risk perceptions and adoption of harm-reducing behaviors, these findings suggest that risk misperceptions are damaging to public health. Persoskie et al. estimated that 370,000 more dual users would have completely switched away from smoking between PATH Waves 2 and 3, had they perceived ENDS to be less harmful than cigarettes [30]. In the current analysis, most Wave 4 smokers thought ENDS were at least as harmful as smoking (74.3%, or 22.5 million smokers), and only 3.3% of these smokers switched at Wave 5. Had these smokers’ risk perceptions been more favorable, and their switch rates accordingly been higher, the analysis suggests that an additional 1.1 million smokers would have switched to ENDS,^1^ with the attendant reduction in exposure to cigarette-smoke related toxicants, [47, 48] and a potential reduction in smoking-related risk [1, 4–6].

Taken together, evidence suggests that correcting smokers’ relative risk perception on ENDS, by providing them with consistent and evidence-based information on relative risks, [49–51] may have a significant impact on their harm-reduction behaviors. As the public misperception of ENDS continues to worsen, educational efforts and interventions are more critical than ever. The role of public health agencies is crucial as a credible source of information for public engagement and communication, as they are one of the most trusted sources of information on the health effects of ENDS [52]. Carefully balanced messages that emphasize the relatively lower risks of ENDS for smokers while also noting the absolute risks for nonusers seem necessary [7, 53].

Aside from the relationship between perceived risk and ENDS use, the PATH data show a steep increase in ENDS adoption and switching in Wave 5, which corresponds to 2019. This is consistent with previously-observed sharp increases in ENDS purchases at this time, [54–56] primarily driven by 4^th^ generation, cartridge-type ENDS [55–57]. These newer generation devices are more satisfying to smokers [58] and are thought to be more effective in inducing complete switching, [59, 60] which may have contributed to the continued decrease in the rate of reversion as well.

Several limitations of these analyses should be taken into account. The observational nature of the PATH study precludes strong causal inferences. The yearly follow-up of the PATH study is not granular enough to capture in detailed behavioral changes between surveys, which makes it challenging to evaluate the precise timing in the changes in behaviors and perception. The interval between surveys varied, but those variations should not be confounded with the variables of interest. The relative risk perception measure was collected in three levels, and the extent of reduced/increased harm was not evaluated. The strongest effect was seen in the contrast between those who perceived ENDS as less harmful than smoking and those who perceived it as more harmful than smoking, though those who perceived the risk of ENDS as equal to that of smoking showed directionally similar effects. One’s risk perception could be affected by various factors, ranging from personal experiences in ENDS use (e.g., physical sensations from use) to publicized changes in major tobacco control policies. The number of switchers included for the reversion analysis was small, which may have resulted in limited power to detect the association between risk perception and reversion among the overall switcher group. No significant interaction effect between risk perception and switching duration in predicting reversion to smoking was found, possibly because the switchers’ association between risk perception and reversion was similar for recent and longer-term switchers. Also, the long-term switchers were very heterogeneous with regard to duration of switching. Longitudinally following a larger sample of ENDS users would be fruitful to address these limitations and may inform the dynamic relationships between risk perception, user experience, and behavioral changes.

Notwithstanding these limitations, this study has significant strengths as well. The analyses were based on large and representative samples of US adult smokers. The analyses considered multiple steps in the process of switching and found directionally consistent effects across them, from the initial adoption of ENDS to complete switching (among current smokers), to reversion from smoking within a year after switching. Factors other than risk perception, such as social influences, also may affect the likelihood of smokers adopting ENDS and switching away from smoking and warrant additional investigation.

## Conclusions

In a nationally representative sample of US adult smokers, perceiving ENDS to be less harmful than cigarettes prospectively predicted adopting ENDS, completely switching to ENDS, and maintaining a switched (non-smoking) status over the first year of switching. As misperceptions about the relative risk of ENDS continues to grow, correcting smokers’ misperception to explain that ENDS are less harmful than smoking could reduce smoking and thereby improve population health.

## Supplementary Information

**Additional file 1:** **Supplementary Table 1.** Associations between three-level risk perception and adoption/switching: results from multivariable GEE modeling. **Supplement Table 2.** Smoking profile and history of participants. **Supplement Table 3.** Sociodemographic characteristics of the sample by risk perception and the proportion of those reporting the outcome in the following wave. **Supplement Table 4.** Sociodemographic characteristics of switchers by switching duration and the proportion of those reporting reversion in the following wave.

## Data Availability

The datasets used for these analyses are from the Population Assessment of Tobacco and Health (PATH) Study; data are available at https://www.icpsr.umich.edu/web/NAHDAP/studies/36498.

## Footnotes

1 Among 22.5 million smokers perceiving equal or more harm of ENDS compared to cigarettes, only 0.7 million smokers (3.3%) reported switching at Wave 5. Had their switching rate been comparable with smokers of more favorable perception (8.0%), the number of switchers in this group would have been as high as 1.8 million, which amounts to 1.1 million additional smokers that could have switched to ENDS.
