# Supplementary material for: US adult smokers’ perceived relative risk on ENDS and its effects on their transitions between cigarettes and ENDS
Source: BMC Public Health. 2022 Sep 19;22:1771. doi: 10.1186/s12889-022-14168-8 (PMC9484256; doi:10.1186/s12889-022-14168-8)
Supplement: Supplementary file 1 — Additional file 1: Supplementary Table 1. Associations between three-level risk perception and adoption/switching: results from multivariable GEE modeling. Supplement Table 2. Smoking profile and history of participants. Supplement Table 3. Sociodemographic characteristics of the sample by risk perception and the proportion of those reporting the outcome in the following wave. Supplement Table 4. Sociodemographic characteristics of switchers by switching duration and the proportion of those reporting reversion in the following wave. [file 12889_2022_14168_MOESM1_ESM.docx]

**Supplementary Table 1. Associations between three-level risk perception and adoption/switching: results from multivariable GEE modeling**

|  | | **Adoption** | **Switching** |
| --- | --- | --- | --- |
|  |  | OR (95% CI) | OR (95% CI) |
| *Time* | Linear | **0.23 (0.12 – 0.44)** | **0.49 (0.29 – 0.83)** |
|  | Quadratic | **1.30 (1.14 – 1.49)** | **1.20 (1.08 – 1.34)** |
| *Risk perception* | More harmful |  |  |
|  | Equally harmful | 1.39 (0.92 – 2.10) | 1.02 (0.62 – 1.68) |
|  | Less harmful | **1.80 (1.17 – 2.75)** | **2.31 (1.45 – 3.67)** |
| *Race/ ethnicity* | NH White |  |  |
|  | NH Black | **0.59 (0.43 – 0.80)** | **0.63 (0.46 – 0.87)** |
|  | Hispanic | **0.56 (0.38 – 0.82)** | **0.72 (0.53 – 0.97)** |
|  | NH others | 1.08 (0.64 – 1.81) | 0.72 (0.51 – 1.02) |
| *Sex* | Male |  |  |
|  | Female | 0.98 (0.76 – 1.28) | **0.85 (0.72 – 1.00)** |
| *Age* | 18 – 24 |  |  |
|  | 25 – 44 | **0.53 (0.37 – 0.76)** | **0.47 (0.37 – 0.60)** |
|  | 45 – 54 | **0.35 (0.24 – 0.52)** | **0.29 (0.20 – 0.42)** |
|  | 55 or older | **0.19 (0.13 – 0.28)** | **0.41 (0.29 – 0.59)** |
| *Marital status*^1^ | Married |  |  |
|  | D/S/W | 1.27 (0.95 – 1.68) | 0.84 (0.65 – 1.09) |
|  | Never married | 1.11 (0.84 – 1.45) | 1.10 (0.87 – 1.39) |
| *Household income* | <$25k |  |  |
|  | $25k – $50k | 0.98 (0.76 – 1.28) | **1.36 (1.06 – 1.74)** |
|  | >$50k | 0.94 (0.70 – 1.27) | **1.62 (1.30 – 2.03)** |
| *Educational attainment* | HS/GED or less |  |  |
|  | Some college | 1.08 (0.85 – 1.37) | **1.54 (1.26 – 1.88)** |
|  | Bachelor or higher | 0.92 (0.63 – 1.33) | **1.87 (1.36 – 2.58)** |

GEE: Generalized Estimating Equations; NH: Non-Hispanic; HS: High school; D/S/W: Divorced/Separated/Widowed
Note: Results are computed from multivariable models that were mutually adjusted for all sociodemographic covariates and time presented here. Boldface represents statistically significant results. The analysis on reversion could not be conducted due to the small number of switchers with a “more harmful” perception.

**Supplement Table 2. Smoking profile and history of participants**

| **Sample characteristics** | | **Smokers who have never used ENDS** | **Smokers who have used ENDS** | **Switchers** |
| --- | --- | --- | --- | --- |
| **Number of observations** | | **N=9331** | **N=23130** | **N=1864** |
| *Average cigarette-per-day*  *(When smoking if switcher)* | | 14.23 (42.1) | 13.95 (20) | 20.18 (27.17) |
| *Years of regular smoking* | | 25.48 (15.3) | 20.24 (14.04) | 19.29 (14.74) |
| *Smoking frequency* | Every day | 7248 (75.3%) | 18004 (78.0%) |  |
|  | Some days | 2083 (24.7%) | 5126 (22.0%) |  |
| *TTFC* | ≤ 30 minutes | 3869 (44.0%) | 9176 (39.5%) |  |
|  | > 30 minutes | 5348 (56.0%) | 13810 (60.5%) |  |
| *Switch history* | < 1 year |  |  | 872 (53.0%) |
|  | ≥ 1 year |  |  | 895 (47.0%) |

TTFC: Time-to-first-cigarette, Switcher: Former smokers who are currently using ENDS
Note: Descriptive statistics presented here are based on all eligible observations collected throughout waves 1 through 4, rather than taking unique participants into account. Participants could report changes in smoking profiles at any given wave and differed in the number of surveys they provided responses to. Data indicate unweighted N (weighted column percentage) for categorical variables and weighted mean (standard deviation) for continuous variables. Boldface represents statistically significant results.

**Supplement Table 3. Sociodemographic characteristics of the sample by risk perception and the proportion of those reporting the outcome in the following wave**

|  | | **Smokers who have never used ENDS** | | | **Smokers who have used ENDS** | | | **Switcher** | | |
| --- | --- | --- | --- | --- | --- | --- | --- | --- | --- | --- |
|  | | *“Less harmful”* | *“Equally or more harmful”* | *Following-wave adoption* | *“Less harmful”* | *“Equally or more harmful”* | *Following-wave switching* | *“Less harmful”* | *“Equally or more harmful”* | *Following-wave reversion* |
| *Race/ ethnicity* | NH White | 1665 (30.7%) | 3597 (69.3%) | 6.1% | 6558 (41.4%) | 9076 (58.6%) | 3.6% | 1221 (87.1%) | 189 (12.9%) | 20.8% |
|  | NH Black | 533 (25.9%) | 1511 (74.1%) | 3.7% | 934 (34.3%) | 1731 (65.7%) | 2.0% | 91 (75.4%) | 34 (24.6%) | 18.2% |
|  | Hispanic | 286 (17.7%) | 1079 (82.3%) | 3.9% | 978 (31.8%) | 1939 (68.2%) | 2.6% | 122 (71.2%) | 52 (28.8%) | 32.2% |
|  | NH others | 146 (24.5%) | 403 (75.5%) | 6.6% | 723 (41.8%) | 1039 (58.2%) | 3.5% | 125 (84.9%) | 26 (15.1%) | 23.4% |
| *Sex* | Male | 1528 (31.1%) | 3275 (68.9%) | 5.2% | 4872 (42.4%) | 6392 (57.6%) | 3.8% | 829 (85.0%) | 152 (15.0%) | 22.9% |
|  | Female | 1125 (23.1%) | 3400 (76.9%) | 5.4% | 4357 (36.3%) | 7508 (63.7%) | 2.7% | 732 (84.4%) | 151 (15.6%) | 20.2% |
| *Age* | 18 – 24 | 261 (34.0%) | 505 (66.0%) | 13.6% | 2339 (43.4%) | 3075 (56.6%) | 5.8% | 264 (75.6%) | 86 (24.4%) | 36.4% |
|  | 25 – 44 | 946 (28.1%) | 2245 (71.9%) | 7.3% | 3948 (39.6%) | 6084 (60.4%) | 3.2% | 694 (85.7%) | 120 (14.3%) | 22.9% |
|  | 45 – 54 | 608 (27.7%) | 1564 (72.3%) | 4.8% | 1464 (38.5%) | 2252 (61.5%) | 1.7% | 258 (87.4%) | 37 (12.6%) | 18.7% |
|  | 55 or older | 827 (26.4%) | 2276 (73.6%) | 2.6% | 1366 (38.3%) | 2201 (61.7%) | 2.4% | 309 (86.0%) | 52 (14.0%) | 14.6% |
| *Marital status^1^* | Married | 978 (28.0%) | 2483 (72.0%) | 4.8% | 2867 (39.1%) | 4380 (60.9%) | 3.2% | 669 (84.3%) | 122 (15.7%) | 17.8% |
|  | D/S/W | 794 (25.8%) | 2155 (74.2%) | 5.0% | 2249 (38.5%) | 3550 (61.5%) | 2.2% | 352 (87.7%) | 55 (12.3%) | 22.3% |
|  | Never married | 872 (29.1%) | 1995 (70.9%) | 6.5% | 4062 (40.5%) | 5853 (59.5%) | 4.1% | 532 (83.4%) | 121 (16.6%) | 27.4% |
| *Household income* | <$25k | 1265 (25.4%) | 3520 (74.6%) | 5.2% | 4063 (35.9%) | 7006 (64.1%) | 2.3% | 429 (81.2%) | 118 (18.8%) | 28.4% |
|  | $25k – $50k | 614 (27.9%) | 1489 (72.1%) | 5.6% | 2210 (40.4%) | 3163 (59.6%) | 3.4% | 412 (84.8%) | 81 (15.2%) | 18.6% |
|  | >$50k | 620 (32.0%) | 1248 (68.0%) | 5.6% | 2464 (44.4%) | 2989 (55.6%) | 4.8% | 640 (87.7%) | 87 (12.3%) | 19.2% |
| *Educational attainment* | HS/GED or less | 1430 (25.0%) | 4149 (75.0%) | 5.0% | 4220 (34.5%) | 7741 (65.5%) | 2.3% | 527 (83.3%) | 131 (16.7%) | 21.1% |
|  | Some college | 825 (29.6%) | 1871 (70.4%) | 6.1% | 3768 (43.1%) | 4934 (56.9%) | 3.9% | 762 (85.4%) | 135 (14.6%) | 23.6% |
|  | Bachelor or higher | 390 (36.1%) | 627 (63.9%) | 5.2% | 1203 (51.1%) | 1153 (48.9%) | 5.4% | 270 (88.1%) | 33 (11.9%) | 17.9% |

Switcher: Former smokers who are currently using ENDS
^1^ Marital status of Wave 1 has been extrapolated from the marital status of Wave 2 due to unavailability in the survey.
Note: Descriptive statistics presented here are based on all eligible observations collected throughout waves 1 through 4, rather than taking unique participants into account. Participants could report changes in their risk perception, smoking/ENDS use statuses and/or sociodemographic factors at any given wave and differed in the number of surveys they provided responses to. Data indicate unweighted N (weighted row percentage). The proportions of outcomes (adoption, switching, reversion) represent weighted percentage of the outcome at the following wave (waves 2 through 5).

**Supplement Table 4. Sociodemographic characteristics of switchers by switching duration and the proportion of those reporting reversion in the following wave**

|  | | **Switch duration: Less than a year** | | **Switch duration: A year or more** | |
| --- | --- | --- | --- | --- | --- |
|  | | *Sample characteristics* | *Reversion in the following wave* | *Sample characteristics* | *Reversion in the following wave* |
| *Race/ ethnicity* | NH White | 663 (77.1%) | 35.9% | 692 (82.3%) | 9.2% |
|  | NH Black | 59 (7.9%) | 24.6% | 55 (5.7%) | 11.1% |
|  | Hispanic | 87 (8.3%) | 44.6% | 67 (6.2%) | 24.2% |
|  | NH others | 84 (6.7%) | 41.1% | 57 (5.8%) | 7.5% |
| *Sex* | Male | 506 (58.3%) | 41.7% | 422 (52.9%) | 26.2% |
|  | Female | 389 (41.7%) | 35.6% | 451 (47.1%) | 9.6% |
| *Age* | 18 – 24 | 245 (19.6%) | 34.4% | 92 (6.5%) | 11.8% |
|  | 25 – 44 | 407 (51%) | 32.0% | 365 (41.9%) | 6.5% |
|  | 45 – 54 | 110 (13.1%) | 37.0% | 167 (20.6%) | 10.8% |
|  | 55 or older | 115 (16.2%) | 34.8% | 226 (31.1%) | 9.3% |
| *Marital status^1^* | Married | 325 (40.4%) | 36.0% | 413 (48.9%) | 6.3% |
|  | D/S/W | 172 (20.8%) | 33.1% | 220 (25.5%) | 13.0% |
|  | Never married | 392 (38.8%) | 38.4% | 233 (25.6%) | 14.5% |
| *Household income* | <$25k | 292 (30.6%) | 39.1% | 228 (26.9%) | 7.4% |
|  | $25k – $50k | 215 (24.8%) | 37.1% | 248 (30.3%) | 13.3% |
|  | >$50k | 344 (44.7%) | 28.5% | 351 (42.8%) | 7.0% |
| *Educational attainment* | HS/GED or less | 330 (35.9%) | 42.8% | 291 (38.6%) | 14.0% |
|  | Some college | 414 (45.1%) | 37.1% | 435 (46.6%) | 6.8% |
|  | Bachelor or higher | 148 (19%) | 31.2% | 145 (14.9%) | 9.5% |

Switcher: Former smokers who are currently using ENDS
^1^ Marital status of Wave 1 has been extrapolated from the marital status of Wave 2 due to unavailability in the survey.
Note: Proportions presented here are based on all eligible observations across waves rather than taking unique participants into account. Participants could report changes in their smoking/ENDS use statuses and/or sociodemographic factors at any given wave and differed in the number of surveys they provided responses to. N indicates number of observations reporting the respective sociodemographic condition, combined across waves 1 through 4, and percentages represent weighted percentage of the outcome at the following wave (waves 2 through 5).
